# PIEZO2 in tumors: from mechanobiological switches to activity-targeted therapies

**DOI:** 10.1186/s13046-025-03598-y

**Published:** 2025-12-01

**Authors:** Dan-xia Huang, Qiu-zhi Zhou, Hong-mei Luo, Mohammad Nasb, Yi-zhou Liu, Yu-jie Yang, Hong Chen

**Affiliations:** 1 Department of Rehabilitation, Tongji Hospital, Tongji Medical College, Huazhong University of Science and Technology, Wuhan, 430030 China; 2 Stem Cell Research Center, Tongji Hospital, Tongji Medical College, Huazhong University of Science and Technology, Wuhan, 430030 China; 3 Rehabilitation Medicine Center/Tuina Department, Provincial Hospital of Traditional Chinese Medicine, Wuhan, 430061 China; 4 Hubei Key Laboratory of Neural Injury and Functional Reconstruction, Huazhong University of Science and Technology, Wuhan, 430030 China

**Keywords:** PIEZO2, Mechanical microenvironment, Cancer prognosis, Therapeutic target

## Abstract

Mechanosensitive ion channel PIEZO2 translates microenvironmental forces into Ca²⁺-dependent signals that shape tumor behavior. Across cancers, PIEZO2 exhibits context-dependent roles, with evidence for both pro- and anti-tumor functions, depending on tissue type, molecular subtype, disease stage, and stromal context. This review synthesizes emerging insights into the multifaceted roles of PIEZO2 in cancer biology, summarize clinical correlations, highlight areas with concordant directionality (e.g., colorectal cancer, medulloblastoma) versus paradoxical findings (e.g., breast cancer across subtypes, gastric cancer by differentiation status), and provide a pragmatic framework for precision, context aware targeting. While PIEZO2 remains attractive for combination strategies (anti-angiogenesis, barrier modulation, dormancy escape, radio/chemo sensitization) and cancer pain interfaces, the field lacks truly PIEZO2 selective small molecule modulators. We propose feasible future priorities encompassing both pharmacologic and non-pharmacologic strategies to accelerate translational applications.

**Graphical Abstract**

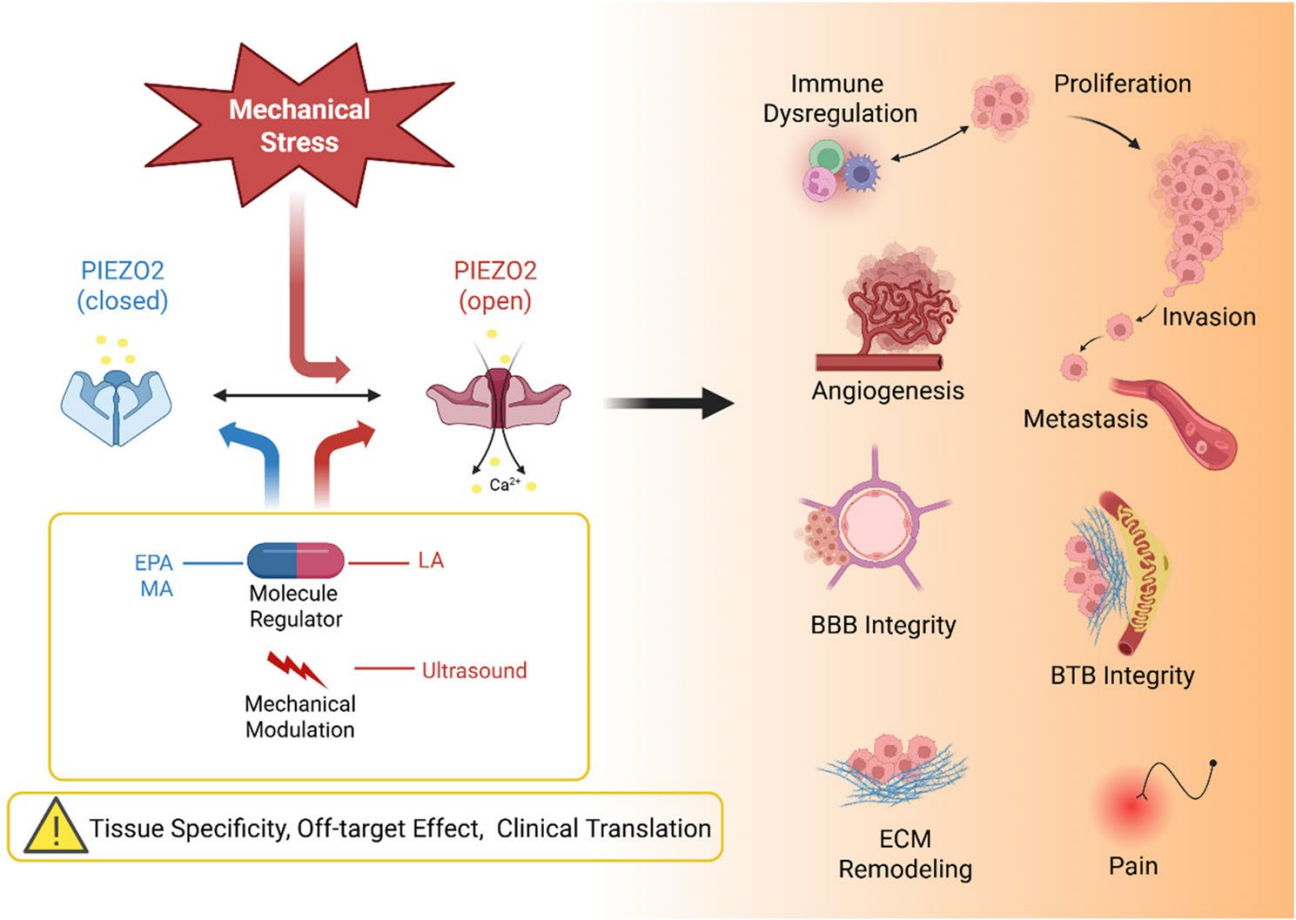

## Introduction

Cancer continues to be one of the leading causes of morbidity and mortality worldwide, affecting millions of people across various populations. It encompasses a diverse range of malignancies that can arise in virtually every tissue and organ of the human body, each exhibiting unique pathophysiological characteristics and responses to treatment [1]. Despite the substantial progress made in early detection, precision medicine, and therapeutic interventions, cancer continues to present significant challenges due to its biological complexity, heterogeneity, and ability to adapt to various hostile environments, underscoring the need for a deeper understanding of cancer biology [2].

Emerging evidence highlights the pivotal role of the tumor microenvironment (TME) in driving cancer progression and therapeutic resistance. The TME is a complex and dynamic ecosystem comprising cellular and noncellular components, including tumor cells, cancer-associated fibroblasts (CAFs), endothelial cells that form microvessels, pericytes, various immune cell populations and the extracellular matrix (ECM) [3]. Previously, this varied assortment of cells was thought to be passive onlookers, mitigating therapeutic effects due to their heterogeneity [4]. However, they are now acknowledged as key contributors to tumor progression, creating a niche for tumor progression and offering promising targets for therapeutic approaches [5].

Within this biomechanical landscape, mechanical forces are increasingly recognized as critical regulators of malignant phenotypes [6–9]. Cells within the TME are constantly exposed to mechanical cues, such as tissue stiffness, fluid shear stress, solid stress, and interstitial fluid pressure (IFP). Such mechanical perturbations are converted into biochemical signals through mechanosensitive signaling pathways, which play essential roles in regulating critical cellular processes, including proliferation, survival, migration, invasion, and metastasis [6].

PIEZO2, a member of the PIEZO family of mechanically activated ion channels, is expressed in tumor cells and stromal cells within tumors. While much of the research on PIEZO2 has focused on its involvement in sensory neurons and mechanotransduction in normal tissues [10–13], emerging evidence suggests that PIEZO2 may also be involved in the pathophysiology and prognosis of cancer [14–17]. By detecting and responding to changes in the mechanical properties of the TME, PIEZO2 may influence key cellular functions, such as cytoskeletal remodeling [18], cell growth [19], and migration [19, 20]. Despite these emerging insights, the precise contributions of PIEZO2 to oncogenic signaling remain incompletely understood.

While several recent reviews have addressed general mechanotransduction or PIEZO1-related cancer signaling [21–24], a comprehensive synthesis dedicated specifically to PIEZO2—its context-dependent roles and translational implications—has been lacking. To address this gap, the present review systematically integrates recent advances spanning molecular mechanisms, cellular phenotypes, and clinical correlations, providing an updated framework for understanding PIEZO2 within the broader mechanobiological network of cancer. In particular, we highlight the potential of PIEZO2 as a combinatorial target al.ongside existing therapies—including anti-angiogenic regimens, chemo- and radiotherapy—and as a unique molecular interface linking tumor mechanobiology to cancer pain modulation. By linking mechanistic discoveries to therapeutic implications, this work aims to define PIEZO2 not merely as a passive mechanosensor, but as a context-aware and targetable nexus in tumor progression and therapy.

## Mechanosensitivity and functional diversity of PIEZO2

PIEZO proteins act as crucial pore-forming subunits within ion channels, responding to diverse mechanical stimuli such as compression, tension, expansion, substrate deflection, and shear stress [25]. The response of PIEZO2 is modulated by both the intensity and the rate of mechanical stimulation, highlighting its sensitivity to dynamic changes in mechanical force [26]. These stimuli induce the release of cations, thereby generating mechanically activated currents across various cell types. This process initiates cellular excitation and triggers essential signaling pathways. Knocking down PIEZO2 resulted in a specific reduction in rapidly adapting (RA) currents (τ_inact_ < 10 ms) [27] and attenuated their inactivation [18]. Notably, PIEZO2 demonstrates nonselective permeability to cations, including Ca^2+^, K^+^, Na^+^, and Mg^2+^, with a notable preference for Ca^2+^ [27, 28].

PIEZO2 is recognized as a polymodal mechanosensor, meaning that it can respond to a variety of mechanical stimuli through distinct force transmission mechanisms. Two predominant models explain the activation of mechanically gated channels in response to mechanical force [29–32]. The force-from-lipids model suggests that membrane tension caused by mechanical forces leads to asymmetry of the transbilayer pressure profile. This alteration induces conformational changes in PIEZO channels, leading to their activation [33, 34]. The force-from-filament model proposes that PIEZO channels are tethered to the cytoskeleton and/or to the ECM and that mechanical forces drive cytoskeletal movements that exert pulling or pushing forces on the channels from the intracellular side, leading to their activation [30]. Both models provide insights into the mechanisms underlying channel activation, but they highlight different aspects of the mechanotransduction process.

## PIEZO2 bridges mechanical forces to tumor biological signaling

**Fig. 1:**
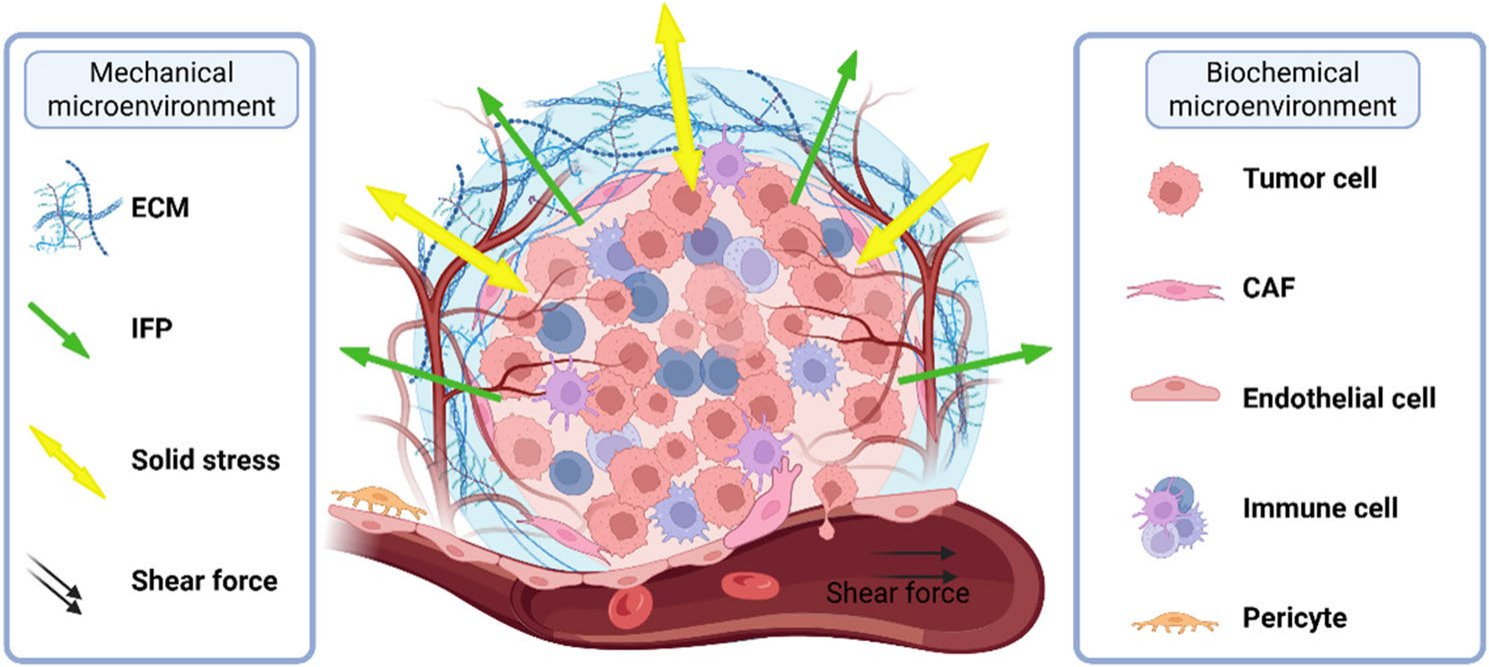
The TME represents a dynamic ecosystem that drives malignant progression by coordinating tumor growth, metastatic dissemination, angiogenesis, and immune evasion through intricate multicellular and molecular crosstalk. These biological processes are profoundly influenced by several interconnected mechanical alterations: ECM stiffening through collagen cross-linking, abnormal shear stress and elevated IFP due to vascular abnormalities, and the progressive accumulation of solid stress from expanding tumor masses. These biomechanical forces collectively constitute a distinctive mechanical landscape that actively shapes tumor behavior. ECM: Extracellular matrix; IFP: Interstitial fluid pressure; CAF: Cancer-associated fibroblasts

The TME is a biomechanically active niche where multidimensional crosstalk between neoplastic cells, stromal populations, and the ECM drives tumor evolution through mechanobiological reprogramming (Fig. 1). The mechanical properties of the TME are not static; they undergo significant changes as the tumor evolves and are influenced by factors such as tumor growth, invasion, and treatment pressures. Mounting evidence has revealed that biomechanical force, spanning ECM stiffening, elevated IFP, and solid stress accumulation, acts as a master regulator of malignant progression.

The ECM plays a pivotal role in the TME, and its dysregulation is a key characteristic of cancer [35]. During tumorigenesis, dynamic interactions between cancer cells and the TME often lead to ECM stiffening [35, 36]. For example, glioma tissues display approximately 10-fold greater rigidity than nonmalignant gliosis tissues, whereas recurrent bladder carcinomas show a 4-fold increase in stiffness relative to normal bladder mucosa [37]. A more pronounced mechanical disparity occurs in mammary malignancies, where tumor stiffness exceeds that of healthy breast parenchyma by nearly 20-fold, with aggressive subtypes exhibiting markedly elevated stiffness values compared with their benign counterparts [35, 38].

Tumor tissues often experience elevated IFP due to ECM remodeling and reduced hydraulic conductivity [39], which is observed across a wide range of tumor types [40]. As tumors grow, their interaction with the surrounding tissue leads to alterations in fluid dynamics within the TME. A significant contributor to these changes is the increase in IFP, which facilitates the movement of interstitial fluid through the tumor stroma [40]. This fluid flow generates shear stress and pressure gradients that impose mechanical forces on tumor cells.

In the TME, the distribution of solid stress exhibits significant spatial heterogeneity. Specifically, within the core region of a tumor, cells are subjected primarily to compressive stresses, whereas at the tumor periphery, cells experience a combination of both tensile and compressive stresses [41]. The rapid proliferation of tumor cells, coupled with the stiffening of the surrounding ECM, contributes significantly to the development of solid stress within the TME [40]. The solid stress in tumor tissues can surpass 150 mmHg (20 kPa) [41].

Solid stress, elevated IFP, and increased ECM stiffness collectively generate heterogeneous mechanical cues in tumors. PIEZO2 responds to membrane tension or deformation by triggering Ca²⁺ influx and downstream signaling [42]. This mechanochemical coupling enables tumor cells and stromal cells to detect changes in mechanical stress and transduce these signals into intracellular biochemical cues, driving hallmark malignant processes encompassing uncontrolled proliferation, apoptosis resistance, metastatic invasion, angiogenic switching, ECM remodeling, immune evasion, and other key processes (Table 1).

**Table 1:** The main signaling pathways regulated by PIEZO2 and their effects on tumors

Signaling pathway	Regulation by PIEZO2	Key downstream biological effects	Tumor effect	Experimental model
Akt/GSK-3β/SNAIL	Enhance	Promoting invasion, migration and metastasis	Pro-tumorigenic	MDA-MB-231 and BT549 breast cancer cells [43]
RhoA/mDia	Enhance	Positively associated with proliferation and migration, matrix degradation, and defense evasion	Pro-tumorigenic	Brain metastatic cells from breast cancer (MDA-MB-231-BrM2) [18]
SLIT2/ROBO1/VEGFC	Enhance	Promoting cell proliferation, migration, invasion and metastasis	Pro-tumorigenic	SW480 colon carcinoma cells [19]
WNT/β-catenin	Enhance	Regulating BTB permeability; Promoting the chemoresistance	Pro-tumorigenic	MB endothelial cell [16, 44]
WNT/β-catenin	Inhibit	Maintaining tumor quiescence	Pro-tumorigenic	Mouse sox2 MB cells; MB mice [16, 44]
Actomyosin/GLUT1	Enhance	Promoting metastasis; Perceiving fluid shear stress	Pro-tumorigenic	Human MB cell lines ONS76, DAOY and D425; Mice bearing ONS76, DAOY or D425 xenograft tumors [45]
SHH signaling pathway	Inhibit	Promoting apoptosis	Pro-tumorigenic	MCF-7 breast cancer cells [46]
JAK2/STAT1/IRF-1	Inhibit	Inducing radioresistance	Pro-tumorigenic	MC38 murine colon adenocarcinoma cells [47]
Ca2+/Wnt11/β-catenin	Enhance	Negatively associated with cell death; Positively associated with cell proliferation, migration, and invasion; Regulating tumor angiogenesis, vascular leakage, and tumor growth	Pro-tumorigenic	GL261 glioma cells; HUVECs; Corneal neovascularization model [48]
Notch signaling pathway	Enhance	Promoting angiogenesis	Anti-tumorigenic	HUVECs [49]

*MB* Medulloblastoma, *HUVECs* Human umbilical vein endothelial cells, *BTB* Blood–tumor barrier

### PIEZO2 as a multifaceted regulator of ECM remodeling

The ECM undergoes continuous biomechanical and biochemical remodeling through the coordinated efforts of stromal and parenchymal cells. Stromal cells, including fibroblasts, endothelial cells, and immune cells, can secrete and degrade ECM components, leading to changes in matrix composition and stiffness [6, 35, 50]. As principal architects of the tumor stroma, CAFs drive interstitial ECM deposition and restructuring, in contrast with basement membrane ECM synthesis by epithelial/endothelial lineages [51]. In the TME, CAFs are dynamic, adaptable, resilient, and engage in intricate interactions with other cell types to drive cancer progression [52, 53]. CAFs are responsible for synthesizing ECM components that provide structural support to the tumor stroma. Moreover, they secrete exosomes, growth factors, and metabolites, which modulate various tumor-related processes, such as the immune response, angiogenesis, and metabolic reprogramming [52, 53]. Emerging evidence has revealed elevated PIEZO2 expression in tumor-associated CAFs [19]. Mechanistic investigations revealed that the expression of PIEZO2 was essential for fibroblast differentiation into profibrotic myofibroblasts and the production of a profibrotic matrix [54]. Specifically, PIEZO2 knockdown significantly suppressed the migration and proliferation of fibroblasts [55], whereas PIEZO2 deficiency attenuated fibroblast activation and fibrotic progression through modulating the activity of mechanosensitive transcription factors and downregulating autophagy-mediated pathways [55].

High expression of PIEZO2 channels markedly enhances ECM synthesis and stability by orchestrating multiple key components of matrix regulation. Specifically, activation of PIEZO2 not only directly promotes the synthesis of fibronectin [54, 56, 57] but also amplifies collagen production signaling [58]. Fibronectin reinforces the ECM architecture by binding to structural proteins such as collagen and integrins, thereby enhancing tissue mechanical stability [36]. Intriguingly, PIEZO2 activation has also been linked to the secretion of Serpin B2, a member of the serine protease inhibitor family [18], which restricts ECM degradation by inhibiting plasminogen activators and reducing plasmin generation [59].

These findings collectively position PIEZO2 as a central molecular hub that integrates mechanical cues with biochemical ECM remodeling in the TME. Notably, the biomechanical consequences of ECM stiffening create a self-reinforcing protumorigenic loop. Increased matrix rigidity potentiates oncogenic signaling pathways [35, 36], underscoring the imperative to investigate mechanotransduction circuits involving PIEZO2 and related channels, as targeting these pathways may disrupt the feedforward relationship between ECM biomechanics and malignant progression.

### PIEZO2 as a mechanotransduction hub orchestrating immune homeostasis

As critical components of the TME, immune cells are recruited to this ecological niche in response to signals emitted by tumor cells [3]. However, the antitumor effector functions of these immune cells are often compromised, leading to a shift toward protumor activities [3]. This alteration can result from various mechanisms, including the secretion of immunosuppressive factors by tumor cells, the presence of regulatory cells, and the dysregulation of cytokine networks [3, 53]. As a consequence, instead of effectively targeting and eliminating tumor cells, immune cells may inadvertently contribute to tumor growth and progression.

Notably, PIEZO2 expression is positively correlated with the infiltration of various immune cell types, including M2 macrophages, CD4 + T memory cells, mast cells, and dendritic cells, within the TME [14, 15]. These immune cell populations are typically associated with tumor progression and an immunosuppressive milieu. The presence of M2 macrophages, known for their role in supporting tumor growth, promoting tumor metastasis and enhancing drug resistance in cancers, may be particularly relevant in this context [60]. Consequently, dysregulation of PIEZO2 expression may contribute to immune dysfunction, thereby suppressing the antitumor response.

The role of PIEZO2 as a mechanotransduction hub in immune homeostasis and tumor progression is an emerging area of research. The roles of PIEZO2 in immune cell infiltration and radioresistance provide a framework for exploring its contributions to immune homeostasis and cancer progression. Further studies are needed to directly link PIEZO2 to immune cell function and tumor mechanics.

### A mechanotransduction hub orchestrating vascular homeostasis and tumor progression

PIEZO2 has emerged as a critical regulator of tumor angiogenesis, and its knockdown shows promising effects on inhibiting tumor growth through antiangiogenic mechanisms [48]. As a critical regulator of embryonic vascular remodeling, an optimal balance of PIEZO2 channel activity is essential for proper vessel formation, structural integrity, and remodeling [61]. Within the tumor vasculature, PIEZO2 is predominantly localized to endothelial cells, where it orchestrates critical angiogenic processes, including endothelial proliferation, migration, and tube formation, through its ability to transduce mechanical cues into intracellular Ca²⁺ signaling. Experimental suppression of PIEZO2 expression has been shown to significantly impede these processes, leading to a significant impairment in the formation of new blood vessels [48, 49]. This disruption deprives tumors of oxygen and nutrients while accumulating metabolic waste, creating a hostile microenvironment that suppresses tumor cell survival and proliferation. Furthermore, PIEZO2 plays a critical role in maintaining endothelial barrier integrity during tumor angiogenesis, acting as an antipermeability factor. PIEZO2 knockdown has been shown to affect vascular permeability [48], further destabilizing the TME. Importantly, the tumor vasculature not only sustains primary tumor growth but also facilitates metastatic dissemination. PIEZO2-regulated vascular permeability and endothelial cell functions underscore PIEZO2 as a central hub that integrates mechanical signals, vascular homeostasis, and tumor progression.

**Fig. 2:**
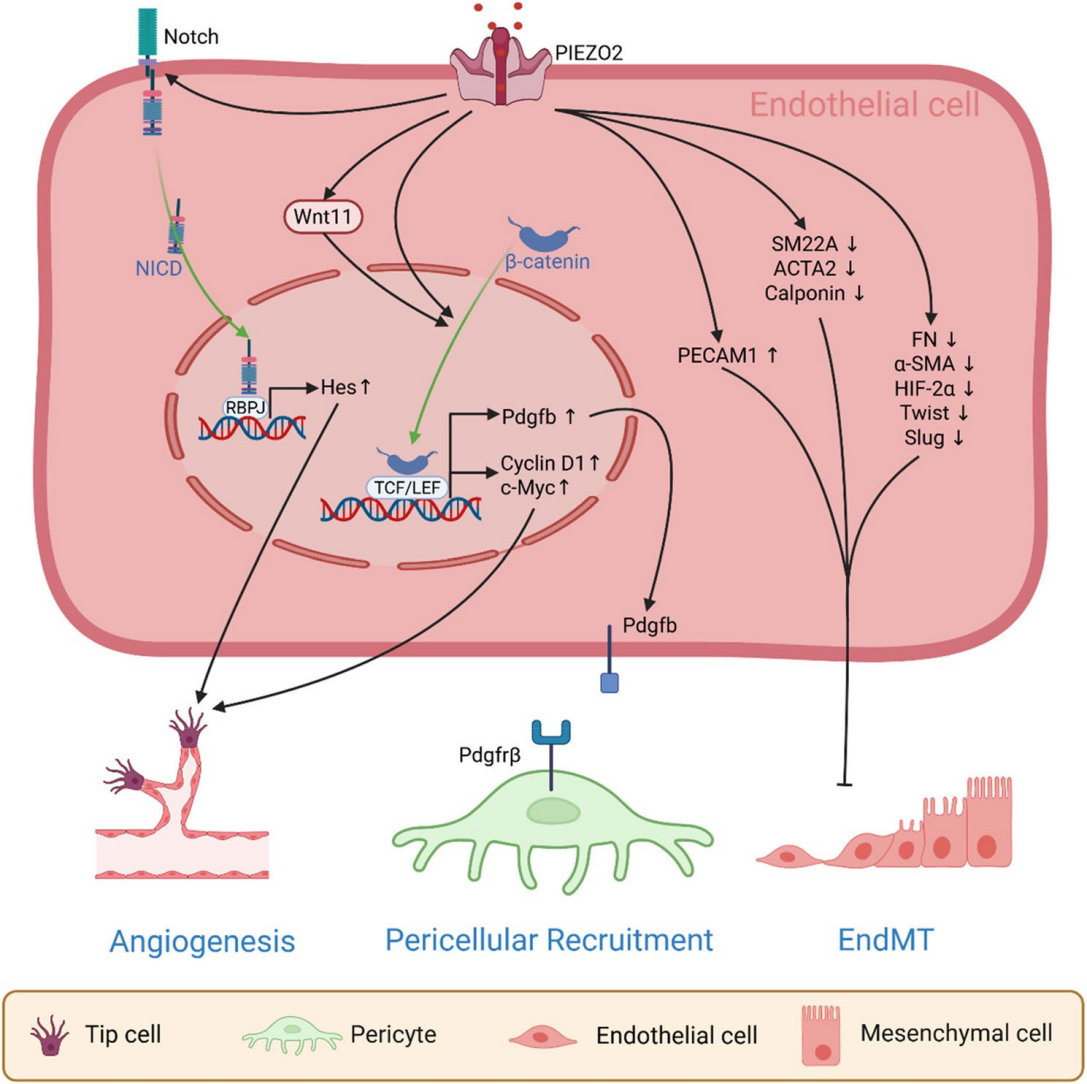
PIEZO2 in endothelial cells regulates tumor angiogenesis, vascular remodeling and pericellular recruitment. EndMT: Endothelial-to-mesenchymal transition; Pdgfb: Platelet-derived growth factor subunit B; Pdgfrβ: Platelet-derived growth factor receptor β; LEF/TCF: lymphoid enhancer factor/T-cell factor

Emerging evidence has revealed that PIEZO2 orchestrates tumor angiogenesis through a multifaceted regulatory network, integrating mechanotransduction with canonical signaling pathways (Fig. 2). PIEZO2 is highly expressed in glioma endothelial cells, where it mediates Ca^2+^ influx and subsequently promotes the expression and secretion of Wnt11. This increase in Wnt11 levels enhances β-catenin stability and facilitates its translocation to the nucleus. β-catenin subsequently associates with LEF/TCF transcription factors to form constitutive complexes. These complexes upregulate key target genes, such as cyclin D1 and c-Myc [48], which are critical downstream effectors of angiogenic signaling pathways. Cyclin D1 promotes cell cycle progression by forming complexes with CDK4/6 [62], whereas c-Myc regulates numerous target genes involved in cell proliferation and metabolism [63, 64]. The upregulation of these genes indicates increased endothelial cell proliferation and angiogenic capacity. Additionally, the downregulation of PIEZO2 expression in human umbilical vein endothelial cells (HUVECs) decreases the expression of Notch pathway-related molecules, including Notch1 (the transmembrane receptor critical for ligand-dependent signaling), Hif1a (a hypoxia-inducible factor that cross-talks with Notch under low oxygen), Hes1 (a critical downstream effector of Notch1 during vascular development [65]), and Mfng (a glycosyltransferase that modulates Notch ligand binding [49, 66]). Activation of Notch involves proteolytic cleavage of the receptor and nuclear translocation of its intracellular domain (NICD) [67, 68]. Within the nucleus, the NICD forms a complex with the DNA-binding protein RBPJ [67, 68]. This NICD/RBPJ complex directly induces the transcription of Hes genes, including Hes1 [67]. The disruption of Notch signaling mediated by PIEZO2 gene knockdown ultimately inhibits the proliferation of endothelial cells and the formation of blood vessels. PIEZO2 also sustains endothelial homeostasis by orchestrating the [Ca^2+^] _I_/phosphorylated SRF/VEGFR2 signaling nexus [69].

Intriguingly, PIEZO2 depletion induces a remarkable phenotypic shift in endothelial cells (Fig. 2). siRNA-mediated knockdown not only reduced the expression of the endothelial lineage marker PECAM1 but also concurrently upregulated the expression of vascular smooth muscle cell (VSMC) markers (SM22A, ACTA2, and calponin) [70]. Moreover, in PIEZO2 knockout endothelial cells, the expression of mesenchymal cell markers (FN and α-SMA) and pro-endothelial-to-mesenchymal transition (EndMT) transcription factors (HIF-2α, Twist, and Slug) is significantly increased [69]. This reciprocal regulation of lineage-specific biomarkers suggests that PIEZO2 maintains endothelial identity by suppressing EndMT [70]. Importantly, EndMT drives tumor progression through multiple mechanisms: providing a cellular origin for CAFs; promoting angiogenesis; enhancing tumor cell proliferation, survival, and metastasis; and facilitating immune evasion [71, 72].

The integrity of the blood‒brain barrier (BBB) and blood‒tumor barrier (BTB) is vital for maintaining central nervous system homeostasis and effectively treating brain tumors. Knockout of PIEZO2 in endothelial cells disrupted WNT/β-catenin signaling, resulting in a marked reduction in the expression of platelet-derived growth factor subunit B (Pdgfb), which subsequently hindered the recruitment of pericytes [16, 44]. These pericytes play crucial roles in maintaining the integrity of the BBB and regulating its functions. These results underscore the critical role of PIEZO2 in maintaining the integrity of the BBB and BTB (Fig. 2).

### PIEZO2 as a multifaceted regulator of tumor homeostasis and prognostic heterogeneity across human cancers

The expression and prognostic implications of PIEZO2 are highly tissue dependent and display remarkable variability among different malignancies (Table 2). The clinicopathological correlations of PIEZO2 expression further underscore its multifaceted biological influences. A comprehensive meta-analysis revealed that PIEZO2 expression is positive association with lymphatic invasion (OR = 7.89, 95% CI: 3.96–15.73) but inverse relationships with invasion depth (OR = 0.17, 95% CI: 0.06–0.47), TNM stage (OR = 0.48, 95% CI: 0.27–0.87), and histological grade (OR = 0.40, 95% CI: 0.21–0.77) [17], reflecting its dual capacity to influence both tumor aggressiveness and differentiation states. Elevated PIEZO2 expression is also significantly linked to lymph-node metastasis and perineural invasion across multiple solid tumors [14, 74–76, 79], reinforcing its functional involvement in mechanosensation-driven metastatic dissemination. These findings collectively highlight the bidirectional and tumor-specific nature of PIEZO2 biology, emphasizing the necessity of context-aware evaluation when considering PIEZO2 as a biomarker or therapeutic target.

**Table 2:** The clinical features of PIEZO2 in various tumors

Tumor	Data source	Tumor effect	Expression level of PIEZO2	Prognosis	Clinical significance	Ref
GC	GC patients	Risk factors	The expression of PIEZO2 mRNA is significantly increased in GC tissue compared to normal tissue (*P* < 0.05).	High expression of PIEZO2 had poor prognosis (HR = 1.48, 95% CI: 1.27–1.69, *P* < 0.0001).	High expression of PIEZO2 positively correlated with survival status (OR = 2.12, 95% CI: 1.31–3.44, *P* = 0.002). High expression of PIEZO2 was significantly linked with Ulixertinib sensitivity (*P* = 0.049, R2 = 0.09).	[73]
	GC patients	Risk factors	/	High expression of PIEZO2 is related to poor prognosis (HR = 2.09, CI: 1.45–3.02, *P* < 0.01). However, it is linked to better prognosis in patients with poorly differentiated GC.	Overexpression of PIEZO2 was associated with T stage (T2 vs. T1, *P* < 0.001; T3, T4 vs. T2, *P* < 0.001), N stage (N2, N3, N4 vs. N1, *P* < 0.05), pathological stage (I vs. III vs. II, *P* < 0.05), histological grade (G1 vs. G2, *P* < 0.01).	[15]
	GC patients	Risk factors	The protein expression levels of PIEZO2 in GC tissues were lower than those in adjacent normal tissues (*P* < 0.05).	Overexpression of PIEZO2 was associated with poor prognosis.	The protein expression level of PIEZO2 was correlated with lymph node metastasis and TNM stage of GC patients (all *P* < 0.05).	[74]
Breast cancer	Breast cancer patients	/	Benign breast tissue demonstrated greater intensity of staining for PIEZO2 than neoplastic tissue (*P* = 0.004) (*n* = 61).	/	A clear relationship was identified between PIEZO2 expression and Ki67 (*P* < 0.05). PIEZO2 expression was associated with perineural infiltration (*P* = 0.01).	[75]
	Breast cancer patients	Risk factors	/	High PIEZO2 expression was linked to worse overall survival in the TNBC cohort (*P* = 0.016), but not in the overall breast cancer cohort or in hormone receptor-positive or HER2-positive subgroups (*P* = 0.784, *P* = 0.555, and *P* = 0.380, respectively).	/	[43]
	Breast cancer patients	Protective factors	The mRNA and protein level of PIEZO2 were decreased in breast cancer tissues compared with normal breast tissues (*P* < 0.05) (*n* = 16).	Breast cancer patients with high expression of PIEZO2 had a significantly favorable prognosis, including OS (HR = 0.72, CI: 0.52–0.99, *P* = 0.04), relapse free survival (HR = 0.61, CI: 0.52–0.72, *P* = 8.9e-10), and distant metastasis free survival (HR = 0.72, CI: 0.52–0.99, *P* = 0.045).	Low PIEZO2 expression was significantly associated with ER status (*P* < 0.001), PR status (*P* < 0.001), HER2 status (*P* < 0.001), T stage (*P* < 0.001), N stage (*P* = 0.0221) and pathologic stage (*P* = 0.0010).	[46]
	Breast cancer cell lines	Protective factors	The expression of PIEZO2 is reduced in breast cancer cell lines (MCF-7, Bcap37, MDA-MB-468 and MDA-MB-231) compared to normal breast cell lines (HBL-100), with a more pronounced decrease observed in high-malignancy cell lines (MDA-MB-468 and MDA-MB-231) relative to low-malignancy cell lines (MCF-7) (All *P* < 0.05)	/	/	[46]
	Breast cancer cell lines	Risk factors	Compared with the parental MDA-MB-231 cells, PIEZO2 was upregulated in the brain metastatic cells from breast cancer (MDA-MB-231-BrM2)(*P* < 0.001).	High expression of PIEZO2 was associated with shorter survival in TNBC patients (HR = 1.73, 95% CI: 1.26–2.98, *P* = 0.04) (*n* = 161).	/	[18]
Colon cancer	Colon cancer patients	Risk factors	The PIEZO2 protein was highly expressed in colon cancer tissues compared with normal samples (18/30 compared to 9/30, χ2 = 5.455, *P* = 0.020).	High expression of PIEZO2 had shorter OS (log-rank (Mantel–Cox) = 15.701, *P* < 0.001).	High expression of PIEZO2 was associated with tumor differentiation (Kendall’s tau-b = 0.360, *P* = 0.002), T stage (Kendall’s tau-b = 0.433, *P* < 0.001), N stage (Kendall’s tau-b = 0.453, *P* < 0.001), and clinical stage (Kendall’s tau-b = 0.322, *P* = 0.006).	[19]
CRC	CRC patients	Risk factors	The expression of PIEZO2 mRNA and protein in CRC tissues was higher than in adjacent tissues (*P* < 0.01). The positive expression rate of PIEZO2 in CRC tissues increases as the degree of differentiation decreases (*P* < 0.01) (*n* = 113).	/	The positive expression rate of PIEZO2 in CRC tissues at TNM stages (I + II) was lower than in those at stages (III + IV) (*P* < 0.01). The positive expression rate of PIEZO2 in CRC tissues with lymph node metastasis was higher than in those without lymph node metastasis (*P* < 0.01).	[76]
Pancreatic cancer	Pancreatic cancer patients	Risk factors	The expression level of PIEZO2 in pancreatic cancer tissues is higher than that in adjacent tissues (χ2 = 25.558, *P* < 0.0001).	The survival curve shows that pancreatic cancer patients with high PIEZO2 expression have a lower OS rate (*P* = 0.032).	The expression of PIEZO2 is related to the depth of tumor invasion (χ2 = 19.155, *P* < 0.05).	[77]
Bladder carcinoma	Bladder carcinoma patients	Risk factors	The expression levels of PIEZO2 in the human bladder cancer group tissues was increased compared to normal tissue (t test; *P* < 0.05).	/	A higher expression level of PIEZO2 in high-grade tumors was detected (pT0 vs. ≥ pT2, *P* = 0.003)	[78]
	Bladder cancer mice	Risk factors	The expression levels of PIEZO2 in the BBN-induced bladder cancer group was higher than in the normal control group (t test; *P* < 0.05).	/	/	[78]
NSCLC	NSCLC patients	Protective factors	The mRNA and protein expression of PIEZO2 in cancer tissue were significantly lower than that in the adjacent noncancer tissues (*P* < 0.05). There was a high gene mutation rate of the PIEZO2 gene in NSCLC.	High expression of PIEZO2 mRNA was correlated to better OS for all NSCLC patients, (HR = 0.54, CI: 0.42–0.69, *P* = 6.4 × 10^− 7^). The high expression of PIEZO2 mRNA was strongly correlated to better OS in LUAD patients (HR = 0.41, CI: 0.28–0.59, *P* = 6.2e-7), but not in LUSC patients (HR = 1.39, CI: 0.88–2.2, *P* = 0.15).	PIEZO2 was associated with worse OS of patients with grades I (HR = 0.38, 95% CI: 0.25–0.57, *P* = 1.1 × 10^− 6^), II (HR = 0.56, 95% CI: 0.32–1.32, *P* = 0.045), and III (HR = 2.52, 95% CI: 1.1–5.78, *P* = 0.024). The low mRNA expression of PIEZO2 was associated with worse OS in patients with negative surgical margins (HR = 0.28, 95% CI: 0.13–0.62, *P* = 0.00079).	[20]
MB	SHH MB patients	Risk factors	/	High PIEZO2 expression was associated with worse survival of SHH MB patients who all had undergone chemotherapy treatment (log-rank(Kaplan‒Meier), *P* = 0.0095) (*n* = 110).	The survival period of *Math1-Cre; SmoM2; PIEZO2*^*+ fl/fl*^ mice treated with etoposide was extended (log-rank(Kaplan‒Meier), *P* = 0.0094) (*n* = 43).	[16, 44]
	SHH MB patients	Risk factors	PIEZO2 is significantly upregulated in tumors from patients (*n* = 390) with SHH MB compared with normal cerebellum tissue sample (*n* = 291).	/	High expression of PIEZO2 is related to metastasis.	[45]
LSCC	LSCC patients	Risk factors	There is significantly higher levels of PIEZO2 promoter methylation in LSCC than normal tissues (*P* = 2.94E-21) (*n* = 99).	PIEZO2 promoter hypermethylation could independently predict a poorer OS (HR = 6.671; 95% CI: 2.087–21.324; *P* = 0.001).	PIEZO2 promoter methylation levels were related to histological classification (*P* = 0.036), T classification (*P* = 0.007), lymph node metastasis (*P* = 0.041), and clinical stage (*P* = 0.006).	[79]

*OS* overall survival, *HR* hazard ratio, *CI* confidence interval, *GC* gastric cancer, *TNBC* triple-negative breast cancer, *MB* medulloblastoma, *CRC* colorectal cancer, *NSCLC* non-small cell lung cancer, *LUAD* lung adenocarcinoma, *LUSC* lung squamous cell carcinoma, *BMCBC* brain metastatic cell from breast cancer, *LSCC* laryngeal squamous cell carcinoma

#### Breast cancer

**Fig. 3:**
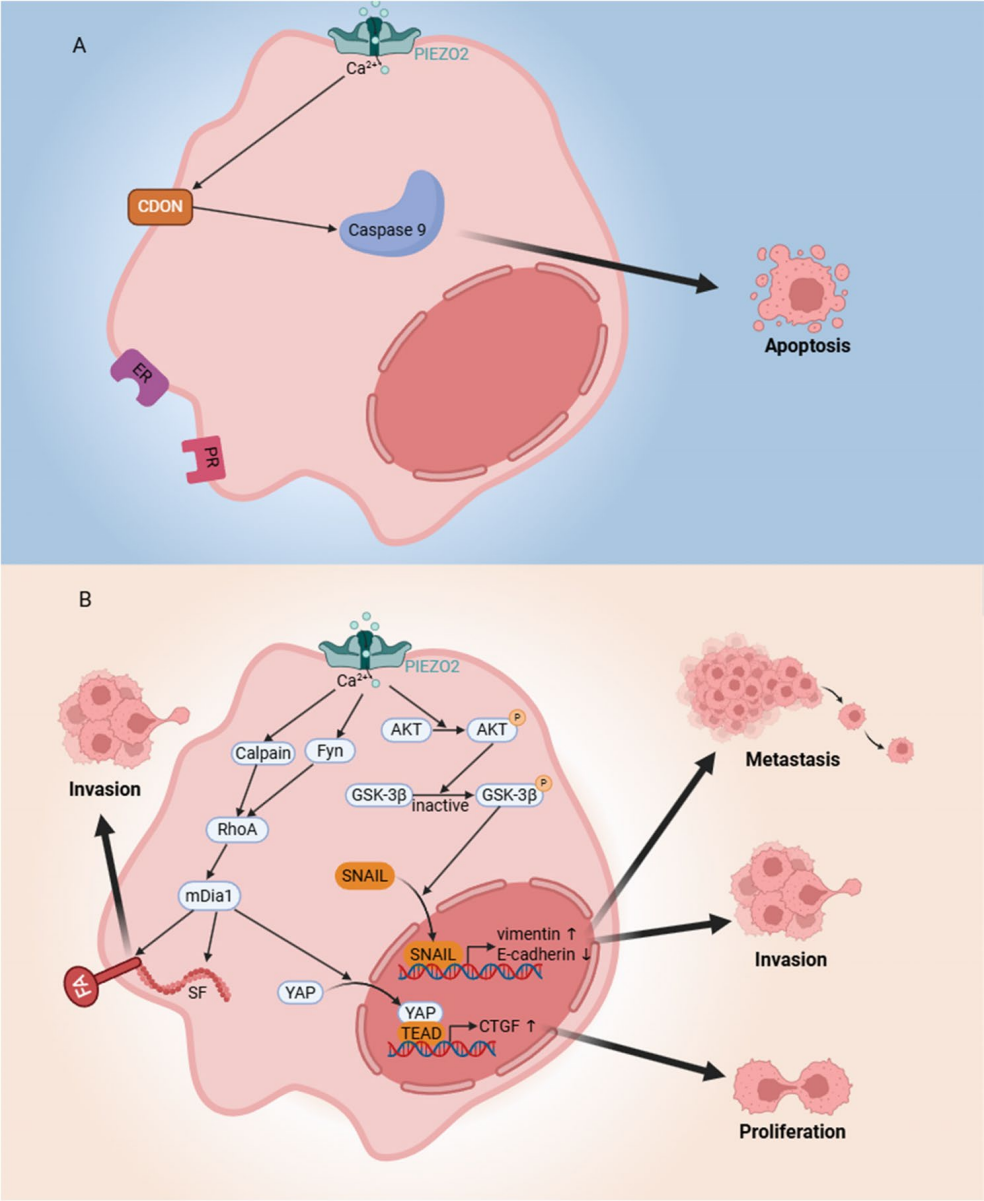
Distinct roles of PIEZO2 in breast cancer subtypes.** A** In ER⁺/PR⁺/HER2⁻ breast cancers, elevated PIEZO2 promotes CDON-mediated caspase-9–dependent apoptosis, suppressing tumor growth. **B** In TNBC (ER⁻/PR⁻/HER2⁻), mechanical activation of PIEZO2 activates Akt–GSK-3β–SNAIL and RhoA–mDia1 signaling, thereby inducing proliferation, invasion, and metastasis. FA: Focal adhesions; SF: Stress fibers; YAP: Yes-associated protein; TEAD: TEA domain transcription factor; CTGF: Connective tissue growth factor; GSK-3β: Glycogen synthase kinase-3beta; CDON: Cell adhesion molecule-related/downregulated by oncogenes

Across breast cancer subtypes, PIEZO2 displays striking heterogeneity and context-specific behavior, reflecting the complex interplay between mechanotransduction and molecular signaling networks (Fig. 3). Immunohistochemical analyses reveal that malignant tissues exhibit relatively uniform but weaker PIEZO2 staining compared with the stronger, interspersed immunoreactivity observed in benign breast tissues [75]. Consistent with this pattern, transcriptomic and proteomic data indicate that PIEZO2 expression is generally downregulated in breast carcinoma relative to normal epithelium [46]. This downregulation correlates with key clinicopathologic markers, including estrogen receptor (ER), progesterone receptor (PR), and HER2 status [46], suggesting that PIEZO2 expression is closely aligned with tumor hormonal signaling profiles.

In ER-positive, PR-positive, HER2-negative, lymph node-positive, luminal A and luminal B breast cancer, higher PIEZO2 levels are linked to favorable clinical outcomes, including prolonged overall and relapse-free survival [46]. This beneficial effect may result from PIEZO2-induced caspase-9–dependent apoptosis mediated by CDON, a Sonic Hedgehog (SHH)-dependent receptor, thereby suppressing tumor growth [46, 80].

However, this protective association does not extend uniformly across all molecular subtypes. In contrast, triple-negative breast cancers (TNBCs) and their brain-metastatic derivatives exhibit an inverse pattern, where high PIEZO2 expression correlates with poor prognosis and enhanced metastatic potential [18, 43]. In TNBC, mechanical stimulation of PIEZO2 triggers Ca²⁺ influx and Akt activation through phosphorylation [43]. Akt-mediated inhibition of GSK-3β stabilizes and drives SNAIL into the nucleus, where it induces vimentin while suppressing E-cadherin expression [43], molecular hallmarks of epithelial–mesenchymal transition (EMT) and metastatic progression.

Recent studies on brain-metastatic breast cancer models have further shown that PIEZO2-mediated calcium influx activates the RhoA and its downstream effector mDia1, which appears to involve recruitment of the Src family tyrosine kinase Fyn to the leading edge of migrating cells, and activation of calpain [18]. The RhoA–mDia1 pathway orchestrates the assembly of actin stress fibers (SFs), alignment of focal adhesions (FAs), and nuclear translocation of YAP [18]. Through these cytoskeletal and adhesive networks, tumor cells generate and transmit traction forces to the ECM, enhancing their migratory capacity. Once in the nucleus, YAP interacts with TEA domain (TEAD) transcription factors to induce target genes such as connective tissue growth factor (CTGF), thereby promoting cell proliferation and tumor progression [18, 81]. Consistent with this, tumors characterized by higher proliferation rates tend to exhibit elevated PIEZO2 scores [75]. Additionally, PIEZO2 demonstrates functional pleiotropy in metastatic progression through its regulation of Serpin B2 secretion [18], which confers dual oncogenic advantages by simultaneously promoting malignant cell survival through apoptosis resistance and potentiating metastatic competence [59, 82].

Taken together, these findings underscore the bidirectional and subtype-dependent roles of PIEZO2 in breast cancer biology. Rather than functioning as a universal oncogenic driver or suppressor, PIEZO2 appears to act as a contextual modulator of mechanical signaling, integrating cellular tension cues with transcriptional programs that dictate tumor phenotype. From a translational perspective, these insights advocate for precision strategies in targeting PIEZO2: inhibition may prove beneficial in aggressive TNBCs, whereas preservation or controlled activation could be advantageous in hormone receptor–positive disease. In particular, combining PIEZO2 modulation with cytoskeletal or EMT-pathway inhibitors may provide a rational avenue for restraining metastasis while respecting subtype-specific dependencies.

#### Medulloblastoma (MB)

In MB, particularly within the SHH molecular subtype, PIEZO2 expression is markedly upregulated in tumor tissues [45]. High PIEZO2 expression is correlated with metastatic potential and poor prognosis [16, 44, 45], and in clinical cohorts of SHH-MB patients receiving chemotherapy, elevated PIEZO2 expression is significantly associated with reduced overall survival [16, 44].

**Fig. 4:**
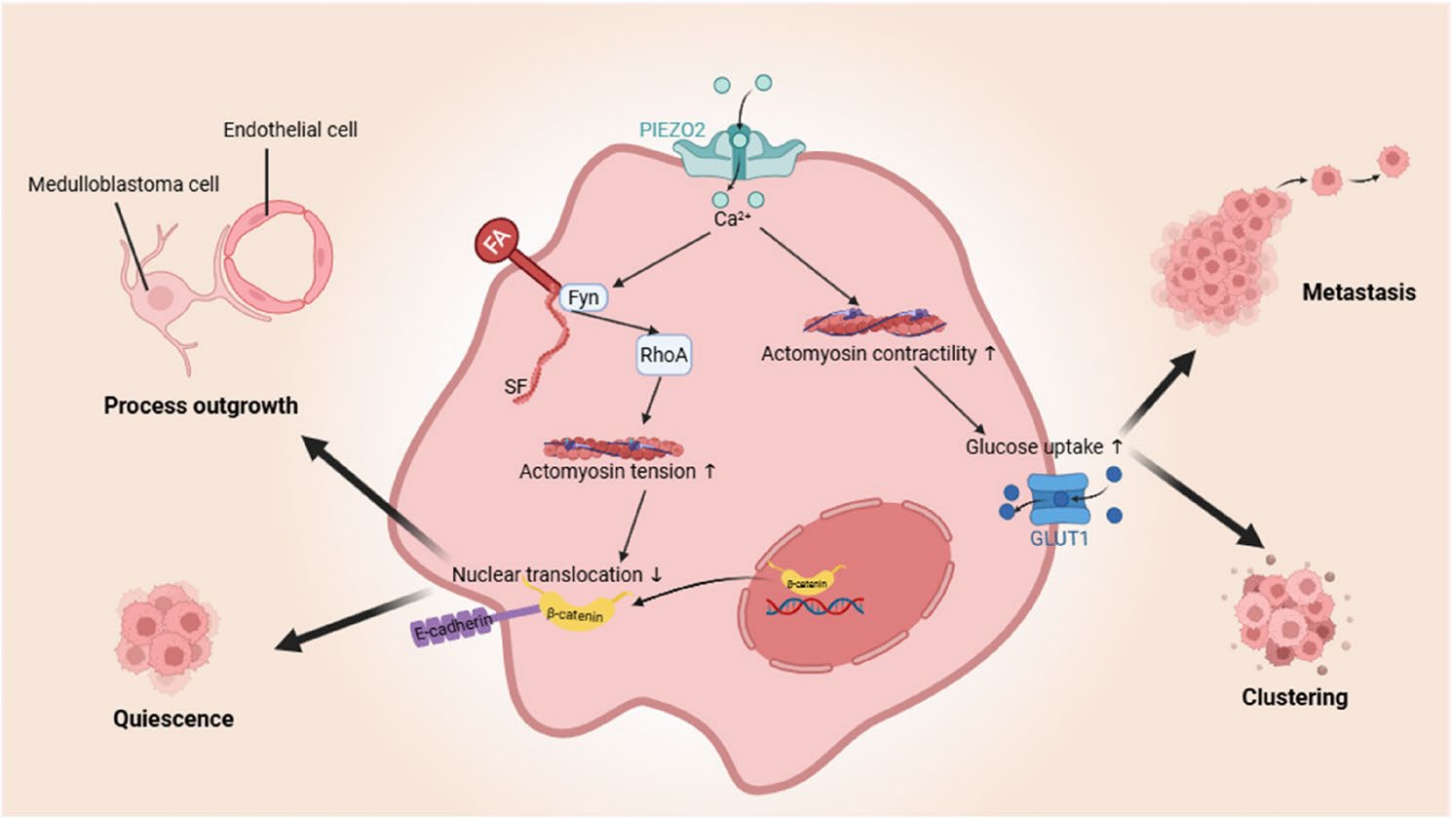
Mechanotransductive pathways regulated by PIEZO2 in MB. PIEZO2 inhibits WNT/β-catenin signaling to maintain tumor cell quiescence and process outgrowth, contributing to a therapy-resistant phenotype, while simultaneously enhancing actomyosin contractility and GLUT1-mediated glucose uptake to promote metastatic behavior. GLUT1: Glucose transporter 1; FA: Focal adhesions; SF: Stress fibers

As a mechanometabolic integrator in MB, PIEZO2 enable tumor cells to sense and adapt to the dynamic mechanical forces of the cerebrospinal fluid microenvironment (Fig. 4). Upon exposure to fluid shear stress, PIEZO2 facilitates mechanosensing in MB cells by inducing Ca²⁺ influx and activating actomyosin contractility [45], a process driven by phosphorylation of myosin light chain 2 (pMLC2). This mechanotransductive signaling cascade promotes the recruitment of glucose transporter 1 (GLUT1) to the plasma membrane and cell–cell junctions, thereby enhancing glucose uptake to sustain tumor cell survival, and motility [45]. Conversely, PIEZO2 knockdown disrupts this adaptive response, leading to diminished GLUT1 localization and impaired cell clustering behavior, a hallmark associated with metastatic potential [45, 83].

In parallel, PIEZO2 activates the Src family kinase Fyn and facilitates its localization to FAs [16, 44]. This triggers SFs formation and RhoA activation, resulting in enhanced actomyosin tension that stabilizes E-cadherin/β-catenin complexes at the plasma membrane [16, 44]. The retention of β-catenin at adherens junctions prevents its nuclear translocation and subsequent activation of Wnt/β-catenin signaling, thereby maintaining cellular quiescence and process outgrowth, which are critical features of a therapy-resistant tumor phenotype [16, 44, 84, 85]. However, despite this regulatory role, genetic ablation of PIEZO2 does not markedly alter MB proliferative output, implying compensatory pathways maintain tumor growth control [16, 44]. Endothelial PIEZO2 additionally maintains BTB architecture by sustaining Wnt/β-catenin activity and Pdgfb expression, which are required for pericyte recruitment and vascular basement membrane integrity [16, 44]. Loss of PIEZO2 weakens the BTB, manifested by thinning of the basement membrane, decreased Lama4 and ColIV, increased Lama2 expression, widened endothelial junctions, and diminished coverage of capillary structures by MB cells, thereby enhancing permeability [16, 44]. In PIEZO2-deficient MB models, drug delivery across the BTB increased more than 19-fold [16, 44]. Inhibition of PIEZO2 synergized with chemotherapy, leading to a marked reduction in tumor burden in preclinical MB models, underscoring its role in mediating treatment resistance.

Through this intricate feedback between mechanotransduction and metabolic signaling, PIEZO2 enables MB cells to flexibly transition between dormancy, invasion, and therapy resistance, positioning it as a promising therapeutic target for disrupting mechanometabolic coupling in SHH-driven MB. Therapeutically, transient or pharmacologic suppression of PIEZO2 represents a promising strategy to enhance drug delivery, overcome dormancy-associated resistance, and disrupt the mechanometabolic–barrier axis that sustains SHH-MB progression. As such, PIEZO2’s roles position it as a compelling target for combination therapies in MB.

#### Colorectal cancer (CRC)

In CRC, PIEZO2 expression is significantly upregulated at both the mRNA and protein levels compared with adjacent non-tumorous tissues [76]. The positive expression rate of PIEZO2 increases progressively with tumor advancement, correlating with poor histological differentiation, higher TNM stage, and lymph-node metastasis [76]. Immunohistochemical analyses reveal that PIEZO2-positive staining is markedly stronger in colon cancer tissues than in normal counterparts [19]. Clinically, elevated PIEZO2 expression is associated with shorter overall survival and with unfavorable pathological features [19], highlighting its potential as a prognostic biomarker in CRC.

Functional studies demonstrate that PIEZO2 enhances colon cancer cell proliferation, migration, and invasion through activation of the SLIT2/ROBO1/VEGFC signaling axis. In contrast, PIEZO2 silencing suppresses these malignant phenotypes and reduces the expression of key pathway components, including VEGFC, SLIT2, ROBO1, and HIF-1α [19], underscoring its regulatory position within this cascade. Slit guidance ligands (SLITs), a family of secreted proteins, signal through Roundabout (ROBO) receptors to mediate interactions between cells and their environment [86]. Activation of the SLIT2-ROBO1 pathway has been shown to enhance tumor cell motility, invasiveness, and metastatic dissemination, primarily through activation of the TGF-β-Smad pathway and HIF-1α/VEGF signaling pathways, and induction of EMT via degradation of E-cadherin [87–90].

Beyond its role in metastasis, emerging evidence implicates PIEZO2 in the modulation of radioresistance and tumor immunity. Radiation exposure markedly increases PIEZO2 expression in colon adenocarcinoma cells, leading to suppression of interleukin-15 (IL-15) production via inhibition of the JAK2/STAT1/IRF-1 axis [47]. Given that IL-15 is pivotal for the activation and maintenance of CD8⁺ T cells—enhancing their effector capabilities and stem cell-like properties while preventing terminal exhaustion—PIEZO2 overactivation may impair antitumor immunity and promote post-irradiation tumor survival [47, 91].

**Fig. 5:**
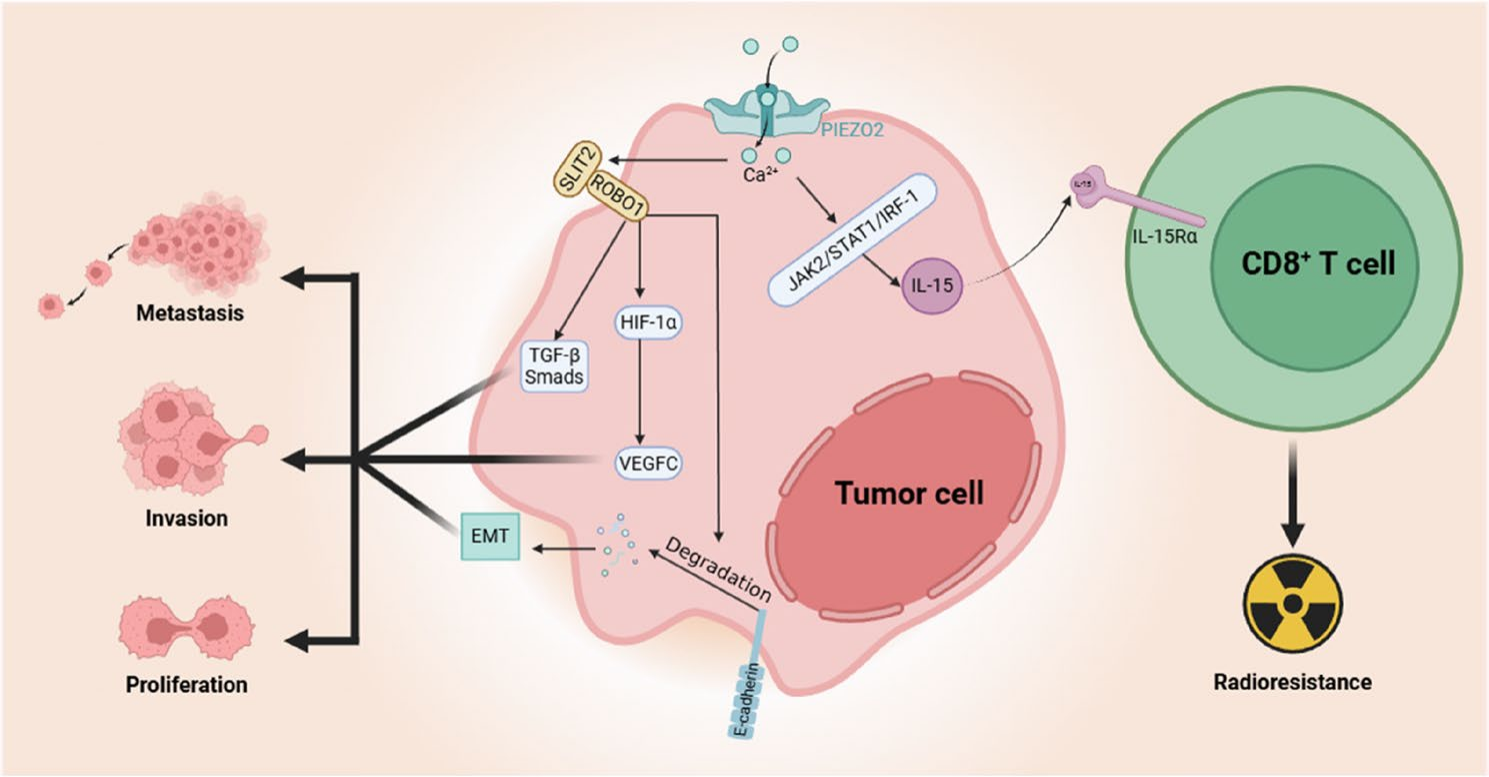
Mechanistic roles of PIEZO2 in colon cancer progression and therapy resistance. PIEZO2 promotes colon cancer cell proliferation, migration, and invasion through activation of the SLIT2/ROBO1 signaling axis, while simultaneously suppressing IL-15 production by inhibiting the JAK2/STAT1/IRF-1 pathway, thereby impairing CD8⁺ T-cell activation and persistence and weakening antitumor immunity. SLIT2: Slit guidance ligands 2; ROBO1: Roundabout 1; IL-15: Interleukin-15; GSK-3β: Glycogen synthase kinase-3beta; EMT: Epithelial–mesenchymal transition

Collectively, these findings position PIEZO2 as a central mechanotransductive regulator in CRC, linking physical microenvironmental cues to oncogenic signaling, and immune evasion (Fig. 5). Targeting PIEZO2 or its downstream signaling partners, while restoring immune activation pathways such as IL-15 signaling, may represent a promising therapeutic approach to counteract metastasis and radioresistance in CRC.

#### Gastric cancer (GC)

In GC, the expression profile and prognostic value of PIEZO2 remain heterogeneous and context dependent, reflecting complex interactions between tumor differentiation, stemness, and the immune microenvironment. Several studies have demonstrated that PIEZO2 mRNA levels are significantly upregulated in GC tissues compared with adjacent normal mucosa, with elevated expression correlating with TNM stages, histological grade, and lymph-node metastasis [15, 73, 74]. Clinically, high PIEZO2 expression has been linked to poor overall survival [15, 73, 74], supporting a potential pro-tumorigenic role. However, other studies report paradoxical findings, noting that higher PIEZO2 expression predicts better outcomes in poorly differentiated tumors [15]. One possible explanation is that PIEZO2 expression is inversely associated with tumor cell stemness and mutational burden in these subgroups [15].

Beyond its direct tumor-cell effects, PIEZO2 also appears to influence the immune landscape of GC. PIEZO2-overexpressing tumors are enriched for immune-related gene signatures, yet paradoxically exhibit a lower predicted response to immune checkpoint blockade, as estimated by the TIDE algorithm [15]. These findings suggest that PIEZO2-mediated mechanical signaling may contribute to immune dysfunction and evasion despite apparent immune cell infiltration [15].

From a translational perspective, PIEZO2 should be viewed not as a uniform prognostic marker but as a dual-function mediator whose influence varies across molecular and histopathologic contexts. Importantly, therapeutic modulation of PIEZO2, either alone or in combination with immune checkpoint inhibitors, may represent a novel strategy to recalibrate immune responses and mechanical signaling in GC. Integrating PIEZO2 expression profiling with mechanical and immune landscape metrics may therefore provide a more precise framework for patient stratification and for predicting responses to immunotherapy.

#### Non-small cell lung cancer (NSCLC)

In NSCLC, the expression of PIEZO2 is significantly reduced in tumor tissues compared with adjacent non-cancerous tissues [14, 20]. However, higher PIEZO2 levels appear to confer a favorable prognostic effect. Across NSCLC cohorts, elevated PIEZO2 mRNA expression correlates with improved overall survival. This association is particularly pronounced in lung adenocarcinoma (LUAD) patients, whereas no significant survival benefit is observed in lung squamous cell carcinoma (LUSC). Notably, PIEZO2 acts as a stronger protective factor in female patients than in males, and its prognostic value diminishes with advancing disease stage, showing greater predictive accuracy in early-stage NSCLC [20].

From a mechanistic perspective, direct functional evidence remains limited, but clinical correlations suggest that PIEZO2 may modulate mechanotransductive signaling linked to tumor differentiation and microenvironmental stiffness. Given its apparent tumor-suppressive association in LUAD, inhibition of PIEZO2 is not advisable. Instead, therapeutic strategies aimed at restoring physiological mechanosensation, such as modulation of ECM tension or vascular microenvironment, may hold translational potential. Collectively, these findings position PIEZO2 as a subtype- and context-dependent protective biomarker in NSCLC, particularly in LUAD and early-stage disease.

#### Other tumors

PIEZO2 dysregulation has also been implicated in several other tumors. In pancreatic cancer, PIEZO2 expression is markedly elevated in tumor tissues compared with adjacent non-tumorous counterparts. High PIEZO2 expression correlates with greater tumor invasion depth and poorer overall survival [77]. Similarly, in human bladder carcinoma, PIEZO2 expression is significantly higher than in normal bladder tissue, and its expression level increases with tumor grade [78]. These findings collectively indicate that PIEZO2 acts as a pro-tumorigenic factor in pancreatic and bladder cancers, potentially facilitating tumor invasion and progression in response to altered mechanical stimuli within the TME. In contrast, in laryngeal squamous cell carcinoma (LSCC), PIEZO2 promoter hypermethylation is significantly more frequent than in normal tissues and serves as an independent predictor of poor overall survival [79]. These findings imply that epigenetic silencing of PIEZO2 may impair normal mechanotransductive signaling, facilitating malignant progression.

Taken together, PIEZO2 exhibits context-dependent oncogenic or tumor-suppressive functions across different cancer types. These mechanistic disparities underscore the importance of tumor context in determining the biological and clinical impact of PIEZO2 and suggest that therapeutic strategies should be tailored to its expression and epigenetic status in each malignancy.

## Context-dependent roles of PIEZO2 in tumors: a paradoxical landscape

PIEZO2 embodies a paradigm of bidirectional functionality in cancer biology and prognosis. Its expression patterns are not uniformly predictive but instead manifest as context-dependent variables capable of indicating either favorable or adverse clinical outcomes on the basis of the specific tumor type, molecular landscape, histological differentiation, and disease stage.

This variability may stem from the extensive diversity of PIEZO2 isoforms, which are driven by cell type-specific alternative splicing mechanisms. In sensory neurons, multiple PIEZO2 splice variants exist and exhibit distinct functional properties, such as varying ion permeability, calcium sensitivity, and inactivation kinetics, enabling precise mechanical sensing [92]. In contrast, nonneuronal tissues predominantly express a single PIEZO2 isoform [92]. In cancer, this splice-dependent functional plasticity likely contributes to the context-dependent roles of PIEZO2, where isoform composition may dictate pro- or antitumorigenic outcomes. Tumors originating from diverse cellular lineages inherit unique splicing landscapes that influence PIEZO2 isoform profiles. Critically, cancer-associated splicing dysregulation may disrupt the physiological balance of PIEZO2 isoforms, potentially through aberrant splicing factors or splice-site mutations altering isoform ratios [93]. Isoforms with enhanced calcium permeability or delayed inactivation kinetics could sustain prolonged Ca²⁺-dependent signaling in aggressive tumors, fueling proliferation, invasion, and therapy resistance. Conversely, truncated or hypofunctional variants may attenuate mechanotransductive signaling, indirectly influencing tumor progression. Furthermore, the mechanically heterogeneous tumor ecosystem, characterized by spatial variations in ECM rigidity, IFP, and shear stresses, may exert selective pressure favoring specific PIEZO2 isoforms optimized for particular biomechanical niches, thereby driving context-dependent adaptations.

The tissue-specific duality of PIEZO2 function also appears to emerge from its dynamic interactions with distinct cellular signaling networks across different microenvironments. For example, in low-malignancy breast cancers, PIEZO2 potentially exerts a protective effect through CDON-Hedgehog axis-mediated induction of proapoptotic signaling, thereby suppressing tumor growth [46]. Conversely, in highly aggressive TNBC, PIEZO2 exhibits oncogenic properties by activating both the Akt/GSK-3β and RhoA-mDia pathways to promote invasive migration [18, 43]. This dual function highlights that PIEZO2 can switch between suppressing or promoting tumors, depending on the specific signals present in the cell.

In addition to direct tumor cell regulation, PIEZO2 influences cancer through effects on nontumor cells within the TME. In immune-rich environments (e.g., GCs), PIEZO2-mediated mechanical signaling in the TME alters immune cell infiltration or activation, thereby facilitating immune evasion [15]. Conversely, in vascular systems, this mechanosensitive ion channel plays a crucial protective role by maintaining endothelial structural homeostasis and resisting pathological remodeling processes [69, 70]. Although not directly linked to cancer pathogenesis, research on pulmonary hypertension (PH) has revealed that PIEZO2 downregulation in endothelial cells could impair the ability of blood vessels to dilate and promote changes in vascular structure, ultimately accelerating PH progression [70]. This vascular-protective mechanism may extend to pulmonary oncology contexts, where preserved PIEZO2 function could inhibit cancer-associated stromal remodeling through mechanisms analogous to its endothelial preservation effects observed in PH pathophysiology, although this remains speculative.

Although genomic alterations in PIEZO2 are relatively rare in tumors, they frequently coexist with other oncogenic mutations, thereby complicating its prognostic interpretation [15]. Moreover, dietary factors [94–96] and inflammatory signals [97, 98] can modulate PIEZO2 expression, suggesting that genetic or environmental regulation varies across cancers.

These collective findings underscore the potential of PIEZO2 as a multifaceted prognostic biomarker with context-dependent clinical implications. Its dualistic roles highlight both therapeutic opportunities and challenges in targeting mechanotransduction pathways across cancer subtypes.

## Forward-looking translational strategies

### Therapeutic strategies targeting tumor progression

The involvement of PIEZO2 in tumor progression and metastasis suggests that targeting PIEZO2 could be a promising strategy for inhibiting cancer spread, particularly for cancers characterized by abnormal mechanical properties and metastasis. Emerging evidence highlights the tumor-suppressive potential of PIEZO2 inhibition across diverse malignancies. PIEZO2 knockdown suppresses proliferation, migration, and invasion while promoting apoptosis in colon cancer cells [19]. Similarly, in breast cancer brain metastasis cells, PIEZO2 knockdown significantly reduces the expression levels of markers associated with cancer invasion and metastasis [18]. Furthermore, PIEZO2 knockdown in endothelial cells has similar inhibitory effects on cellular proliferation and motility but also attenuates the invasive capacity of cocultured glioma tumor cells through antiangiogenic mechanisms and antipermeability [48]. These collective findings suggest that strategic PIEZO2 inhibition could simultaneously target multiple hallmarks of cancer. This multimodal therapeutic approach holds particular promise for addressing the dual clinical challenges of primary tumor eradication and metastatic niche prevention, potentially overcoming the limitations of conventional monotherapeutic strategies.

### Pain management in therapy

Cancer pain is frequently observed in patients, especially in advanced stages of the disease [99]. Cancer-related pain predominantly arises from cancer secretory factors and tumor invasion of pain-sensitive tissues, including bone, soft tissue, nerves, viscera, and blood vessels, and can also be induced by therapeutic interventions such as surgery, chemotherapy, or radiation. PIEZO2 has been shown to be involved in the development of sensitized mechanical pain following both inflammation and nerve injury [100–103]. The Epac1-PIEZO2 signaling pathway in the DRG may play a role in the processing of mechanical allodynia signals during the development of bone cancer pain [104]. Moreover, the clinical anticancer drug vincristine induces mechanical allodynia and hyperalgesia through a PIEZO2 channel-dependent mechanism [105]. Chemotherapeutically induced peripheral neuropathic pain is a prevalent side effect of chemotherapy, often limiting the administration of optimal dosages for effective cancer treatment [106, 107]. Injection of ODN antisense agents targeting PIEZO2 mRNA significantly reduced mechanical hyperalgesia in a model of oxaliplatin chemotherapy-induced painful peripheral neuropathy [108], highlighting its translational potential. In this context, targeting the inhibition of PIEZO2 channels could represent a promising analgesic strategy for tumor treatment. By inhibiting PIEZO2, clinicians could mitigate pain without exacerbating opioid dependence, thereby improving quality of life and enabling adherence to curative therapies.

### Integrating mechanobiology into combination therapies

The development of chemotherapeutic resistance remains a significant challenge in cancer treatment, particularly in tumors with a strong tendency for dormancy or those shielded by physical barriers [109, 110]. Recent studies have shown that the BTB in MB is mechanically reinforced through PIEZO2-dependent signaling. Genetic knockout of PIEZO2 in MB cells has been shown to disrupt this mechanical integration, reducing tissue rigidity and enhancing drug delivery across the BTB. Furthermore, PIEZO2 ablation diminishes the quiescent phenotype of tumor cells, sensitizing them to chemotherapy by disrupting their dormancy-associated survival mechanisms. Preclinical studies have demonstrated that PIEZO2 inhibition synergizes with chemotherapy, significantly reducing the tumor burden in MB models [16, 44]. These findings collectively position PIEZO2 as a promising therapeutic target to overcome chemoresistance in tumors in specific contexts, such as MB.

In addition to its role in chemoresistance, emerging data suggest that PIEZO2 might contribute to an immunosuppressive TME. There is a negative correlation between PIEZO2 expression and CD8 + T-cell infiltration, as well as the radiosensitivity of rectum adenocarcinoma patients undergoing radiotherapy [47]. High levels of PIEZO2 in tumor cells are associated with an immune-suppressive environment, which is correlated with impaired CD8 + T-cell responses and reduced efficacy of radiotherapy [47, 91]. These findings suggest that PIEZO2-targeted therapies may convert immunologically “cold” tumors into “hot” tumors, thereby potentially expanding the applicability of immune checkpoint inhibitors and adoptive T-cell therapies. However, direct experimental evidence for this conversion in vivo is currently lacking.

The potential role of PIEZO2 in mediating resistance to conventional therapies, including chemotherapy and radiation, is an area that requires further exploration. It is possible that PIEZO2-mediated mechanotransduction could contribute to tumor cell survival under therapeutic stress, suggesting that combined therapies targeting PIEZO2 and other key signaling pathways may provide increased therapeutic benefits. Rational therapeutic options could include the following: (1) PIEZO2 inhibitors with cytotoxic agents to bypass stroma-mediated resistance and (2) PIEZO2 inhibitors with radiotherapy to overcome immune exclusion and improve radiotherapy sensitivity. The efficacy of these combinations requires rigorous validation in diverse preclinical models and clinical settings.

### Mechanical modulation of PIEZO2 for tumor treatment

PIEZO2 can be activated by various mechanical stimuli [25]. This sensitivity to mechanical cues positions PIEZO2 as a potential target for noninvasive interventions. Ultrasound technology has emerged as an ideal modality for such interventions, offering three-dimensional spatial precision (millimeter-scale resolution) in delivering focused acoustic energy to deep anatomical targets while maintaining noninvasive advantages [111]. The biomechanical effects induced by ultrasound waves, encompassing both thermal and mechanical bioeffects, create unique opportunities for targeted tissue modulation at substantial depths. Although direct evidence that ultrasound activates PIEZO2 in cancer is limited, studies in other contexts have demonstrated that ultrasound-induced pressure waves can activate mechanosensitive ion channels [112, 113] and stimulate PIEZO expression [114], suggesting a plausible pathway for PIEZO2 activation.

Therapeutic applications could exploit the bidirectional regulation of PIEZO2 in malignancies: low-intensity pulsed ultrasound (LIPUS)-mediated mechanical preconditioning may therefore serve to restore mechanotransduction homeostasis in PIEZO2-deficient tumors, potentially reversing malignant phenotypes associated with mechanosensing impairment. Conversely, high-intensity focused ultrasound (HIFU) could leverage the mechanoamplification properties of PIEZO2 to achieve selective tumor ablation, where malignant cells with channel overexpression exhibit increased susceptibility to ultrasound-induced mechanical disruption.

Recent advancements in ultrasound-responsive bioengineering have expanded these therapeutic horizons. For instance, the application of therapeutic focal ultrasound has been shown to noninvasively enhance the anti-cancer efficacy of tumor necrosis factor–related apoptosis–inducing ligand (TRAIL) in prostate cancer by mechanically activating mechanosensitive ion channels within tumor cells [113]. Additionally, the Wang laboratory has pioneered an innovative ultrasound-responsive mechanogenetic system that leverages PIEZO ion channels to detect ultrasound-induced mechanical perturbations and subsequently transduces these physical signals into programmable genetic activation [115]. When integrated with chimeric antigen receptor (CAR) T-cell therapies, this technology enables remote-controlled immunomodulation—ultrasound waves serve as both targeting signals (through PIEZO-mediated spatial recognition) and activation triggers (via mechanogenetic induction of therapeutic transgenes), establishing a novel paradigm for precision oncotherapy [115].

### Pharmacological strategies for targeting PIEZO2 in tumor therapy

For modulation of PIEZO2, current practice relies on non-selective blockers of mechanosensitive channels, lipid‑membrane modulation, cytoskeletal axis drugs, or biologics in discovery (nanobodies/peptides).

Classical non-selective blockers of mechanosensitive cation channels, including ruthenium red and gadolinium, suppress PIEZO2 currents but suffer from poor selectivity and translational limitations [116]. Peptidic inhibitors such as the spider-venom toxin GsMTx4 reversibly inhibit PIEZO2 by modulating membrane tension coupling [116], serving as a mechanistic scaffold for future small-molecule optimization. Lipid–protein interactions provide an alternative pharmacological avenue, as phosphatidic acid (PA) has been identified as an endogenous negative regulator of PIEZO2 gating [117], and modifications of membrane composition by cholesterol [118], phosphatidylinositol-4,5-bisphosphate (PIP₂) [119], or polyunsaturated fatty acids [94–96] can fine-tune channel mechanosensitivity. Beyond membrane lipids, cytoskeletal dynamics have emerged as a critical regulatory layer influencing PIEZO2 mechanogating. Disruption of the actin cytoskeleton with cytochalasin D or latrunculin A [26, 94, 95, 101, 105], stabilization with jasplakinolide [26, 95], or inhibition of microtubule polymerization by vincristine [105] or colchicine [101] markedly alters PIEZO2 activation thresholds, underscoring the channel’s strict dependence on cytoskeletal integrity and force transmission through interconnected actin–microtubule networks. Pharmacological modulation of cytoskeletal regulators—such as Rho GTPases, focal adhesion kinase (FAK), or actin-binding adaptors—thus offers an indirect yet tractable route to tune PIEZO2 activity without directly occluding the ion-conducting pore. Inflammatory signaling cascades, notably those mediated through the bradykinin B2 receptor–PKA/PKC axis [120], further potentiate PIEZO2 activation, suggesting that kinase modulators could indirectly desensitize the channel under pathological conditions. Accessory proteins such as Nedd4-2 [121], TMEM120A (TACAN) [117, 122], and TMC7 [123] have also been shown to negatively regulate PIEZO2, offering additional indirect targets for drug discovery.

Particularly, dietary fatty acids, known for their “natural, low-side-effect, and synergistic benefits,” could provide a safer complementary approach to traditional cancer treatments. A diet enriched with safflower oil, which is high in linoleic acid (LA), significantly enhances the stability and function of PIEZO2. This dietary intervention has demonstrated a notable therapeutic effect, effectively reducing the progression of hypoxia-induced PH [69] and improving gait ataxia in a mouse model of Angelman syndrome (AS) [95]. Additionally, a diet enriched with eicosapentaenoic acid (EPA) has been shown to reduce the activity of PIEZO2, which could help alleviate joint defects in mice with gain-of-function (GOF) mutations in PIEZO2 [96]. Given their proven therapeutic effects in other PIEZO2-related diseases [69, 95, 96], these fatty acids also show potential for use in cancer therapy, especially in relation to PIEZO2 modulation. For example, margaric acid (MA) and EPA have demonstrated therapeutic potential by suppressing PIEZO2 activity [94, 96], thereby providing a possible means to inhibit PIEZO2-mediated oncogenic processes, including tumor cell proliferation, invasion, angiogenesis, and metastasis. Conversely, LA has been shown to augment PIEZO2 activity [95], suggesting a potential strategy for targeting malignancies characterized by PIEZO2 deficiency, such as NSCLC. This bidirectional modulation underscores the importance of tailoring lipid-based interventions to tumor-specific PIEZO2 expression levels.

## Overcoming challenges in PIEZO2-targeted cancer therapies

Despite significant advances, several key limitations impede the development of PIEZO2-targeted therapies for cancer.

1. Substantial uncertainty persists regarding the specific roles of distinct PIEZO2 isoforms and splice variants. The functional consequences of alternative splicing, isoform-specific expression patterns, and their tissue-specific contributions to mechanotransduction remain poorly understood, complicating the development of targeted interventions. Thus, comprehensive single-cell multiomics analyses (transcriptomics, proteomics, and spatial transcriptomics) are needed to map isoform-specific expression patterns and their associations with tumor subtypes and TME niches within clinical samples. CRISPR-Cas9-based functional genomics (e.g., isoform-specific knockouts/knock-ins in relevant cellular models) can elucidate the oncogenic or tumor-suppressive roles of individual variants. The development of isoform-selective tools, such as antisense oligonucleotides or siRNAs that target unique splice junctions, subsequently offers a pathway for functionally specific intervention.
2. The dualistic nature of PIEZO2, which functions as both an oncogenic accelerator and a tumor suppressor, complicates its categorization as a universal biomarker or therapeutic target. Interpreting the prognostic importance of PIEZO2 or evaluating its potential as a therapeutic target necessitates a nuanced, mechanobiology-informed approach. The development of effective therapeutic strategies must be rooted in a comprehensive understanding of the complex mechanisms underlying the actions of PIEZO2 across different cancer types. Future translational efforts should focus on elucidating the context-specific roles of PIEZO2, as well as identifying biomarkers that can predict its effects in individual tumors.
3. Although this review primarily emphasizes the central role of PIEZO2 in cancer progression and prognosis, it is equally important to acknowledge the intrinsic complexity of tumor mechanobiology. Several studies have reported that in cells where PIEZO2 is knocked down or deleted, mechanosensitive currents and mechanosensitive functions, is attenuated but not eliminated [16, 18, 45, 124]. This observation strongly suggests the presence of PIEZO2-independent mechanosensory mechanisms operating within the dynamically heterogeneous TME. For instance, PIEZO1, a closely related homolog, can mediate similar Ca²⁺ influx and has been implicated in tumor growth, angiogenesis, and migration [23, 125]. Likewise, TRPV4 and TRPM7 channels contribute to EMT [126, 127], whereas TRPV2 promotes cancer progression through the activation of autophagy [128]. Moreover, integrin-mediated signaling, the transcriptional coactivators YAP and TAZ and the cytoskeletal network, can independently sense and transduce mechanical cues [129–132]. Therefore, it is more accurate to view PIEZO2 as a key node rather than the sole executor within the broader mechanotransduction network of cancer. A major future challenge will be to delineate the relative contribution, interaction, and compensatory relationships among these distinct mechanosensitive pathways under specific tumor types and microenvironmental conditions. Understanding this redundancy is crucial for developing effective anticancer strategies, as it implies that targeting a single channel (e.g., PIEZO2) may yield limited benefit, whereas combined inhibition of multiple mechanosensory pathways may provide more robust therapeutic outcomes.
4. A major pharmacological hurdle is the lack of highly specific, potent, and pharmacologically tractable modulators (both agonists and antagonists) for PIEZO2. The development of such selective modulators is exceptionally challenging owing to the high structural homology and conserved gating mechanisms shared between PIEZO1 and PIEZO2. Innovative strategies to overcome this include exploring cryo-EM-derived high-resolution structures for structure-based drug design (SBDD) that target PIEZO2-specific allosteric sites or regulatory domains distinct from those of PIEZO1. The development of proteolysis-targeting chimeras (PROTACs) is promising for achieving the catalytic, isoform-selective degradation of PIEZO2, potentially minimizing off-target effects. Additionally, discovery campaigns for biologics (e.g., nanobodies or engineered peptides) that target unique extracellular epitopes on PIEZO2 could yield highly selective agents. Furthermore, potential cross-reactivity with other structurally unrelated mechanosensitive channels must also be considered. Collectively, these converging strategies define the emerging pharmacological landscape of PIEZO2.
5. Efficiently delivering therapeutic agents to the specific TME where PIEZO2 dysregulation occurs presents another significant obstacle. Factors such as the physicochemical properties of potential PIEZO2 modulators (e.g., size, charge, hydrophobicity), the dense and often abnormally stiff ECM characteristic of many solid tumors, and the need for precise spatial targeting to avoid systemic toxicity complicate delivery strategies. Given the importance of PIEZO2 in normal physiological processes, such as mechanosensation and tissue homeostasis, strategies that selectively target its aberrant activity in cancer cells while minimizing off-target effects on healthy tissues are crucial. Emerging strategies focus on advanced delivery platforms, particularly nanocarriers for localized delivery, to overcome ECM barriers and achieve precise spatial targeting. Furthermore, nonviral delivery systems (e.g., lipid nanoparticles) for PIEZO2-targeted siRNA or gene editors offer alternative modalities for localized gene silencing.
6. Current knowledge relies heavily on in vitro systems and murine models. Although invaluable, these models may not fully recapitulate the complexity of human PIEZO2 function, signaling pathways, or disease pathophysiology. For example, standard 2D culture fails to recapitulate the complex mechanical microenvironment (stiffness, confinement, fluid shear) crucial for PIEZO2 activation. Enhanced model fidelity can be achieved through the development of advanced humanized mouse models and the implementation of sophisticated 3D culture systems.
7. A pronounced lack of clinical trials exists for therapies specifically targeting PIEZO2. While preclinical studies suggest therapeutic potential, translating these findings into safe and effective human treatments remains unvalidated. Bridging this substantial gap between promising preclinical results and demonstrated clinical efficacy remains a crucial hurdle. High-throughput screening using large-scale libraries of patient-derived tumor organoids offers a human-relevant platform for predictive efficacy and toxicity profiling. To complement these preclinical advancements, exploratory phase I basket trials enrolling patients with diverse PIEZO2-dysregulated solid tumors should be initiated to assess safety and identify preliminary efficacy signals. The proposed trial designs incorporate biomarker stratification, utilizing PIEZO2 expression levels (via RNA sequencing or immunohistochemistry) combined with ECM stiffness quantification (e.g., atomic force microscopy ex vivo, ultrasound elastography in vivo) to identify responsive patient populations and evaluate safety/efficacy.

## Conclusions

The mechanosensitivity and functional diversity of PIEZO2 enable it to respond to various mechanical stimuli, thereby influencing a wide range of cellular processes. In the context of tumor progression, PIEZO2 acts as a multimodal mechano-regulatory hub. Its mechanosensitivity enables dynamic responses to ECM stiffness, IFP, fluid shear stress, and solid stress, modulating tumor cell behavior, the TME, and even tumor–nerve interactions. However, the roles of PIEZO2 in tumors are highly context dependent, presenting a paradoxical landscape in which it can either promote or inhibit tumor progression depending on the specific cellular and environmental context. This paradox highlights the necessity of dissecting microenvironmental determinants, such as mechanical stress, neural interactions, and immune landscapes, that shape PIEZO2 activity and downstream effects.

Targeting PIEZO2 activity holds promise for cancer treatment. Despite the absence of specific PIEZO2-targeted activators or inhibitors, its activity and expression can be indirectly modulated through interconnected pathways, suggesting novel strategies for cancer intervention. Several potential targets and strategies have been identified to modulate its function. However, forward-looking translational strategies must address these challenges through precision approaches that account for tumor subtype, stage-specific mechanosignaling, and dynamic TME mechanics. The therapeutic potential of PIEZO2 lies not in broad suppression but in precision targeting informed by the tumor subtype and biomechanical profile of the TME. Future research must unravel the molecular logic governing the paradoxical roles of PIEZO2 and translate these insights into mechanomedicine paradigms that harmonize efficacy with physiological safety. However, despite promising findings in preclinical models, the translation of PIEZO2-based therapies to clinical settings remains an area of uncertainty. More robust clinical trials, along with comprehensive basic research, are needed to fully understand the therapeutic implications of targeting PIEZO2, including its safety, efficacy, and long-term outcomes.

## Data Availability

No datasets were generated or analysed during the current study.

## Abbreviations

TME: Tumor microenvironment
ECM: Extracellular matrix
IFP: Interstitial fluid pressure
CAFs: Cancer-associated fibroblasts
RA: Rapidly adapting
YAP: Yes-associated protein
FA: Focal adhesions
SF: Stress fibers
GLUT1: Glucose transporter 1
pMLC2: Phosphorylation of myosin light chain 2
IL-15: Interleukin-15
HUVECs: Human umbilical vein endothelial cells
VSMC: Vascular smooth muscle cell
EMT: Epithelial–mesenchymal transition
EndMT: Endothelial-to-mesenchymal transition
BBB: Blood–brain barrier
BTB: Blood–tumor barrier
TGF-β: Transforming growth factor-beta
GSK-3β: Glycogen Synthase Kinase-3beta
CDON: Cell adhesion molecule-related/downregulated by oncogenes
ROBO: Roundabout
SLIT: Slit guidance ligand
TEAD: TEA domain transcription factor
CTGF: Connective tissue growth factor
LEF: Lymphoid enhancer factor
TCF: T-cell factor
Mfng: Manic fringe
SHH: Sonic Hedgehog
Pdgfb: Platelet-derived growth factor subunit B
Pdgfrβ: Platelet-derived growth factor receptorβ
OS: Overall survival
NSCLC: Non-small cell lung cancer
MB: Medulloblastoma
PNI: Peripheral nerve invasion
ADC: Antibody‒drug conjugates
PH: Pulmonary hypertension
TNBC: Triple-negative breast cancer
BMCBC: Brain metastatic cells from breast cancer
GC: Gastric cancer
CRC: Colorectal cancer
LUAD: Lung adenocarcinoma
LUSC: Lung squamous cell carcinoma
LSCC: Laryngeal squamous cell carcinoma
LIPUS: Low-intensity pulsed ultrasound
HIFU: High-intensity focused ultrasound
TRAIL: Tumor necrosis factor–related apoptosis–inducing ligand
CAR: Chimeric antigen receptor
PA: Phosphatidic acid
PIP₂: Phosphatidylinositol-4,5-bisphosphate
FAK: Focal adhesion kinase
LA: Linoleic acid
AS: Angelman syndrome
EPA: Eicosapentaenoic acid
GOF: Gain-of-function
MA: Margaric acid
SBDD: Structure-based drug design
PROTACs: Proteolysis-targeting chimeras
